# Oxidative Stress in Cognitive and Epigenetic Aging: A Retrospective Glance

**DOI:** 10.3389/fnmol.2020.00041

**Published:** 2020-03-18

**Authors:** Aditi Kandlur, Kapaettu Satyamoorthy, Gireesh Gangadharan

**Affiliations:** Department of Cell and Molecular Biology, Manipal School of Life Sciences, Manipal Academy of Higher Education, Manipal, India

**Keywords:** oxidative stress, brain aging, learning and memory, cognitive decline, epigenetic aging, molecular mechanisms

## Abstract

Brain aging is the critical and common factor among several neurodegenerative disorders and dementia. Cellular, biochemical and molecular studies have shown intimate links between oxidative stress and cognitive dysfunction during aging and age-associated neuronal diseases. Brain aging is accompanied by oxidative damage of nuclear as well as mitochondrial DNA, and diminished repair. Recent studies have reported epigenetic alterations during aging of the brain which involves reactive oxygen species (ROS) that regulates various systems through distinct mechanisms. However, there are studies which depict differing roles of reactive oxidant species as a major factor during aging. In this review, we describe the evidence to show how oxidative stress is intricately linked to age-associated cognitive decline. The review will primarily focus on implications of age-associated oxidative damage on learning and memory, and the cellular events, with special emphasis on associated epigenetic machinery. A comprehensive understanding of these mechanisms may provide a perspective on the development of potential therapeutic targets within the oxidative system.

## Introduction

Aging involves the systemic loss of functioning in a time-dependent manner (Childs et al., 2015). There is increased focus on deciphering the mechanisms that prevail during aging. This is due to the increase in the life expectancy of individuals over these past few decades. The World Population Prospects 2019 report states an increase in the average life expectancy at birth to 72.6 years in 2019. The same report indicates that by 2050, the global number of individuals aged 65 years and more, will surpass the number of youths aged 15 to 24 years. The number of older individuals is projected to grow more than double the number of children below 5 years of age, by 2050 (United Nations, 2019). Aging is a key factor in several neurodegenerative disorders such as Alzheimer’s and Parkinson’s diseases (Hindle, 2010; Uddin et al., 2018). An aging brain is reported to show more cases of amyloid plaques, α-synuclein accumulation, loss of brain volume, loss of neurons, and neurofibrillary tangles (Elobeid et al., 2016). The hippocampus and the frontal lobes are the most commonly affected brain regions, therefore bringing about observable phenotypes pertaining to cognition, learning, memory, and attention (Peters, 2006). Brain being a metabolically robust active organ, is a large consumer of oxygen as well as producer of reactive oxygen species (ROS) (Halliwell, 1992). Although there are many theories to explain the mechanism of aging, the free radical theory forms a basis to explain a great deal about how the reactive oxidants interact with the components of the cells to bring about a long-lasting accumulated damage and result in aging (Harman, 1968; Sohal and Brunk, 1992; Cutler and Mattson, 2006; Oswald et al., 2018). There seems to be a converging viewpoint regarding the epigenetic machinery that would be influenced by the changes in the oxidative microenvironment of cells. However, with the role of a double-edged sword, reactive oxidant species proves to be a necessary evil which also has been shown to be involved in various essential functions of the neuronal cells such as polarization of neurons, neurite outgrowth and axon specification, activity-induced synaptic growth and plasticity (Oswald et al., 2018). This review will highlight the roles played by the oxidative environment in the cells as aging proceeds and how it affects learning and memory as well as its significance in neurodegenerative diseases. The review will also include the underlying cellular events with an emphasis on the associated epigenetic machinery.

## Oxidative Stress and Cognitive Aging

Cognition is the collective set of abilities that involves various information processing, storage, and retrieval. This envelopes the concept of intelligence or metacognition, which involves the capacity to learn from experience and the ability to adapt to the surrounding environment or situations (Sternberg and Sternberg, 2011). There is a visible deterioration in cognition in aged individuals, in terms of fluid abilities or intelligence. This involves the application of crystallized abilities (skills and memories that are acquired) based on context/situation. These cognitive abilities that decline with age include attention, language skills, executive cognitive function, visuospatial abilities (perceiving spatial orientation of objects and visuoperceptual judgment), and certain types of memory such as working memory, prospective memory (to remember to perform a task in future) among others. There is also a well-defined and gradual decline in learning capacities (Critchley, 1984; Lezak et al., 2012; Murman, 2015). There are variations in the course of cognitive aging due to genetic as well as environmental factors. Such factors affect the cell’s characteristic resilience to accumulation of damage that are brought about by stressors of aging (Barter and Foster, 2018). In terms of memory, episodic memory is the most effected with aging. This type of memory would include the memories of events in a spatio-temporal context (where and when it occurred) and with aging, retrieval becomes more difficult. On the other hand, semantic memory that covers memory of general knowledge of vocabulary, facts, etc. which does not require quick retrieval actions are retained quite well (Levine et al., 2002; Kinugawa et al., 2013). The hippocampus of the limbic system is the critical focal point or structure for the cognitive aspects such as acquiring information (learning), consolidation and recall of declarative (episodic and semantic) memories (Eichenbaum, 2001). It is an important region concerned in the spatial memory maps formation (Papp et al., 2007). The loss of memory retention and recall may be due to age dependent loss of hippocampal functioning. The memory in the hippocampus is presumed to be time-limited, in the manner that it stores memories fast and also undergoes faster overwriting, this in turn might represent a higher decay rate of such aspects of memory. However, the remote memory remains to be integrated into the neocortical regions, with the ventromedial prefrontal cortex becoming a major sector for memory consolidation in the limbic system-cortex axis (Nieuwenhuis and Takashima, 2011; Talamini and Gorree, 2012). This decline in cognitive abilities that accompanies aging may be partly due to brain atrophy that occurs in the temporal and frontal regions (Peters, 2006; Castelli et al., 2019).

Oxidative stress and its associated damage being involved in the age-dependent cognitive loss have been highlighted through numerous investigations to be the basis of pathogenesis. Comparison between young and aged animal brains showed higher levels of ROS and oxidative stress markers (Sohal et al., 1994; Liu et al., 2003; Serrano and Klann, 2004). The appearance of behavioral deficits or cognitive impairments in temporal and spatial memory, learning and retention of memory displayed strong associations with increasing oxidative species and stress in aged animal models (Carney et al., 1991; Fukui et al., 2001).

The conditions that prevail during aging are higher levels of oxidant species, oxidative stress and a shortfall in the antioxidant levels. To counter the effects of drop in antioxidant levels, the aged mice models that overexpress extracellular superoxide dismutase (EC-SOD) showed alleviation in spatial learning and memory related hippocampal long-term potentiation (LTP) that declines with age (Hu et al., 2006). They also showed better working memory as well as retention capacities when compared to the aged wild-type mice (Kamsler et al., 2007). On the other hand, to counter the effects of ROS, an antioxidant rich diet was able to reverse metabolic deficiencies of ascorbic acid, arachidonic acid, α-tocopherol. It even decreased the age-associated increase in the SOD activity, lipid peroxidation as well as inflammatory cytokine IL-1β. This might suggest the oxidative stress due to changes in the cellular environment might have an impact on the LTP (O’Donnell and Lynch, 1998). Age-associated cognitive impairment in terms of object recognition and associative fear memory has been observed in the *klotho* mouse model of aging. These mice showed higher levels of oxidative damage in lipid and DNA in the hippocampus and cerebral cortex. They also showed increased activity of antioxidants Cu/Zn-SOD and glutathione peroxidase (GPx) to compensate the increased ROS levels, the hippocampus also showed higher apoptotic gene expression. These mice when treated with α-tocopherol showed recovery of memory and reduced lipid peroxidation as well as cell death in the hippocampus, indicating oxidative stress caused apoptosis which later progressed into memory impairment (Nagai et al., 2002). A similar study with aging rats and vitamin E deficient rats showed decreased learning as well as memory retention ability whereas younger rats supplemented with vitamin E displayed accelerated learning and capabilities. This could be that the learning ability declined gradually during aging due to chronic exposure to oxidative stress. The decrease in the memory retention capacity was due to oxidative stress induced delayed type of apoptosis that was observed in the CA1 hippocampal region. Vitamin E administration ameliorated the memory deficit. The vitamin E deficient rats showed higher levels of thiobarbituric acid reactive substances (TBARS), lipid hydroperoxides and protein carbonyls in the synaptic membranes. Synaptic membranes showed decreased ζ-potential causing a deficit in depolarization of the membrane and causing faulty neurotransmission (Fukui et al., 2001). Another study highlighted increased oxidative stress with aging in terms of increased TBARS with corresponding increase in Cu/Zn-SOD and mitochondrial Mn-SOD activity. The aged mice also displayed impaired mitochondrial electron transfer in certain complexes- complex I and III (brain NADH-cytochrome c reductase), complex IV (brain cytochrome oxidase) and brain citrate synthase. The study describes that neurodegeneration and longevity can be predicted through the neuromuscular functioning and the oxidative stress markers. It highlights the existence of a relationship between the oxidative stress and the behavioral performance in terms of exploration (Navarro et al., 2002). In the case of apoE knockout studies, it has been observed that there is a combined influence on cognitive aging through systemic oxidative stress and inflammatory vascular dysfunction (Evola et al., 2010). The age-related impairment of cognition involved oxidative molecular damages in the brain being one of the factors implicating cortical involvement. The same study established that the functional associations fall short to establish causal links that oxidative damage alone is able to cause age associated cognitive decline (Forster et al., 1996). Oxidative stress causing damage to mitochondria has been recently been implicated in neuronal degeneration as well as decline in cognition in Alzheimer’s subjects (Reddy, 2006). In case of implicated neurons in Alzheimer’s, oxidative damage is brought about by the free radicals generated by the entry of mutant APP and soluble Aβ into mitochondria and cause further impair mitochondrial metabolism (Manczak et al., 2006). Mild cognitive impairment (MCI) patient samples that precedes Alzheimer’s shows a vast amount of oxidative damage in the brain in terms of lipid peroxidation, protein carbonyls, and malondialdehyde (Keller et al., 2005). Another study showed a strong correlation between decreased antioxidant levels and increased lipid peroxidation, also stating that the depleted antioxidant systems may be the end result of the increased oxidant species levels (Padurariu et al., 2010). Large scale cohort studies in humans such as EVA revealed a strong link as to oxidative stress being involved in cognitive decline with aging (Berr et al., 2000).

## Cellular and Molecular Implications of Oxidative Aging

A prominent sign of an aging cell is the imbalance between the constantly produced reactive oxidant species and the diminishing antioxidant capacity (Halliwell, 1992). To have a better outlook on the role of oxidative stress during normal aging as well as disease conditions such as neurodegenerative disorders that accompany aging, we here discuss various levels of implications imposed by aging in the oxidative microenvironment- cellular, organellar, genetic as well as epigenetic.

### Oxidative Damage Sources and Their Implications

DNA is an important carrier of heritable genetic information that faces the limitation of chemical stability that is constantly prone to changes (Lindahl, 1993). Considering the large genome size and the slower rate of replication when compared to the prokaryotes, it can be estimated that human cells have a turnover rate of 2,000–10,000 DNA purine bases on a daily basis due to hydrolytic depurination followed by excision repair. In this context, it has been hypothesized that long-lived, non-dividing human nerve cells would lose ∼10^8^ purines or ∼3% of its total purine residues from its DNA in an individual’s lifetime (Lindahl and Nyberg, 1972).

DNA undergoes oxidative damage due to a series of sources reactive oxygen and nitrogen species (RONS), reactive carbonyl species, products of lipid peroxidation (De Bont and van Larebeke, 2004). The ROS are superoxide (O_2_^–⋅^), singlet oxygen (^1^O_2_), hydrogen peroxide (H_2_O_2_), and hydroxyl radicals (OH^⋅^). Reactive nitrogen species include nitrous anhydride (N_2_O_3_) and peroxynitrite (ONOO^–^). They bring about deaminating reactions on guanosine and adenosine (Burcham, 1999) (see Table 1).

**TABLE 1:** Oxidizing species – their targets and products.

Oxidizing species (source)	Target	Oxidative damage product	References
Superoxide anions	Guanine	5-Diamino-4*H*-imidazolone (Iz) and 8-oxo-7,8-dihydroguanine (8-oxoG)	Misiaszek et al. (2004)
Singlet oxygen	Guanine	8-Oxo-7,8-dihydroguanine and spiroiminodihydantoin	DeFedericis et al. (2006)
Hydroxyl radicals	Adenine/adenosine	5-Formamido-6-aminopyrimidine type product (FAPy) adenine and adenosine; 8-hydroxyadenine or -adenosine	Steenken (1989)
	Cytosine	5-Hydroxy-5,6-dihydrocytos-6-yl and 6-hydroxy-5,6- dihydrocytos-5-yl	Chabita et al. (1996)
	5-Methylcytosine	5,6-Dihydroxy-5,6-dihydro-5-methylcytosine; 1-carbamoyl-4,5-dihydroxy-5-methyl-2-oxo-imidazolidine; aminocarbonyl[2-amino]-carbamic acid and *N*-formamide and 4-amino-1-5-dihydro-5-methyl-2-*H*-imidazol-2-one	Bienvenu and Cadet (1996), Bienvenu et al. (1996)
Nitrous anhydride	Adenine	Hypoxanthine	Burney et al. (1999)
	Cytosine	Uracil	
	5-Methylcytosine	Thymine	
	Guanine	Xanthine	
Peroxynitrite	Deoxyguanosine	8-Nitro-deoxyguanosine	Douki and Cadet (1996), Burcham (1999)
	Deoxyadenosine	8-Oxo-7,8-dihydro-2′-deoxyadenosine	Douki and Cadet (1996)
	Guanine	8-Nitroguanine	Love (2006)

#### Level of DNA Breaks With Oxygen Tension

Oxidative damage causes profound effects on the genetic composition by affecting the nuclear and mitochondrial DNA. The relative amounts of such damages if quantified can give an idea regarding the levels of oxidative stress faced by the cell, especially during aging. This has been done previously by various groups using different techniques such as HPLC-EC or GC/MS (see Table 2).

**TABLE 2:** Relative amounts of oxidative damages on nucleic acids in aging.

Type of oxidative damage	Rate of production	Sample studied	Rate of repair required/hits on DNA	Technique used to measure oxidative damage	Species	Age groups	Source of oxidative stress	References
8-Hydroxydeoxyguanosine (8OHdG)	236 fmol/μg of DNA	Liver	165 ± 66 pmol kg^–1^ day^–1^	HPLC- electrochemical detection	Rat	24 months	Naturally occurring	Fraga et al. (1990)
	37.5 ± 3.2 fmol/μg of DNA	Kidney						
	16.7 ± 1.1 fmol/μg of DNA	Intestine						
	13.1 ± 2.5 fmol/μg of DNA	Brain						
	13.2 ± 0.9 fmol/μg of DNA	Testes						
	3.2 residues/10^6^ bp	Liver	20% cleavage per μg DNA	HPLC- electrochemical detection	Mouse	4 months	Naturally occurring	Klungland et al. (1999)
	8–73 per 10^6^ dG residues	Liver	Not mentioned	HPLC- electrochemical detection	Rat	6 months	Naturally occurring	Richter et al. (1988)
8-Hydroxyguanosine (8OHG)	3645 ± 1166 pmol kg^–1^ day^–1^	Urine	Not mentioned	HPLC- electrochemical detection	Rat	24 months	Naturally occurring	Fraga et al. (1990)
8-Oxoguanine (8-oxoG)	76.2 ± 6.15 nmol/mmol creatinine	Urine	Not mentioned	HPLC and GC/MS	Rat	14 months	Naturally occurring	Foksinski et al. (2004)
	84.99 ± 5.91 nmol/mmol creatinine		34,000 repair events/cell/day		Mouse	12 months		
	8.4 ± 1.21 nmol/mmol creatinine		2,800 repair events/cell/day		Human	40 years		
8-Oxo-deoxyguanosine(8-oxodG)	0.037 ± 0.004 per 10^5^dG residues	Liver	47,000 lesions/cell/day	HPLC- electrochemical detection	Mouse	4–8 months	γ-Irradiation (0.5–50 Gy	Hamilton et al. (2001)
	0.012 ± 0.003 per 10^5^dG residues	Brain	Not mentioned					
	0.012 ± 0.004 per 10^5^dG residues	Heart	Not mentioned					
	0.033 ± 0.005 per 10^5^dG residues	Liver	Not mentioned		Rat	4–6 months	Naturally occurring	
	0.012 ± 0.003 per 10^5^dG residues	Brain	Not mentioned					
	0.010 ± 0.002 per 10^5^dG residues	Heart	Not mentioned					
	0.064 ± 0.004 per 10^5^dG residues	Prostate	Not mentioned		Human	60–78 years	Naturally occurring	
8-Oxo-deoxyguanosine(8-oxodG)	7.22 ± 1.05 nmol/mmol creatinine	Urine	Not mentioned	HPLC and GC/MS	Rat	14 months	Naturally occurring	Foksinski et al. (2004)
	13.2 ± 1.23 nmol/mmol creatinine		34000 repair events/cell/day		Mouse	12 months		
	2.1 ± 0.44 nmol/mmol creatinine		2800 repair events/cell/day		Human	40 years		
8-Oxo-deoxyadenosine(8-oxodA)	59 per 10^5^ nucleosides	Aqueous solution of DNA	Not mentioned	HPLC- electrochemical detection	–	–	Peroxynitrite solution	Douki and Cadet (1996)

### Cellular and Organellar Changes

The aging cell displays certain nucleocytoplasmic features which describe the events that precede and that follow the oxidative damage in various organelles as well as other subcellular compartments. As a part of the normal respiration, ROS such as superoxide is produced from the oxygen consumed, these reactive species interact with iron–sulfur clusters and release free iron, which triggers the downstream release of more reactive oxidant species (Boveris, 1984). Hydrogen peroxide from superoxide produce highly reactive hydroxyl radical that drive the oxidative damage toward the DNA, lipids, and proteins (Halliwell and Gutteridge, 1985). Mitochondria is a major internal source for ROS and hence is also a major target of oxidative damage (Chance et al., 1979); progressive impairment of mitochondria has been implicated in aging and neurodegenerative disorders such as Alzheimer’s (Swerdlow and Khan, 2004; Mecocci et al., 2018). The mitochondrial DNA (mtDNA) is vulnerable to the insults of ROS as they lack histones. The mtDNA has been reported to show increasing levels of oxidized nucleoside 8-hydroxy-2′-deoxyguanosine (OH8dG) with oxidative damage that gradually increases with aging and has been observed in Alzheimer’s as well (Mecocci et al., 1993, 1994). Apart from mitochondrial DNA damage, models of premature aging also shows a disruption in DNA repair through defective repair proteins such as Ku86 as well as impairment of transcription-coupled repair of RNA polymerase II stalling lesions (Vogel et al., 1999; De Boer et al., 2002). The age-associated oxidative stress may be a common pathogenetic factor in the neurodegenerative disorders that show occurrence of cytoplasmic aggregates, which may be due to p62 and cytokeratins accumulation and aggregation with misfolded proteins (Zatloukal et al., 2002). In case of comorbidities of Alzheimer’s such as Idiopathic normal pressure hydrocephalus, there have been reported mitochondria-endoplasmic reticulum contact (MERC) sites. These sites are said to be in a feedback-loop type regulation, wherein these sites can regulate the amyloid β-peptide (Aβ peptide) and the peptide can regulate these sites. These MERC sites were reported to increase in patients with dementia (Leal et al., 2018). Lysosomes exhibit increased accumulation of partially digested or damaged matter carried from the inhibited proteasomal activity. Proteasome inhibition also brought about an increase in the levels of mitochondrial ROS, loss of mitochondrial homeostasis and led to increased autophagy that might lead to brain aging (Sullivan et al., 2004). This has been observed in pathological conditions of Alzheimer’s, where there is accumulation of soluble Aβ peptide in the lysosomes (Ditaranto et al., 2001). The damage by oxidative stress is seen to build up on nuclear pores. It alters nucleocytoplasmic transport as it affects nuclear lamina components. Aging also caused loss of nuclear pore protein such as Nup93. Aged neurons of rats possessed leaky nuclei and also accumulation of intranuclear tubulin bIII which caused severe chromatin aberrations (D’Angelo et al., 2009). Phosphorylated tau which is characteristic of Alzheimer’s alters the transport across the nucleocytoplasmic axis. This was shown to occur by direct interactions with a nuclear pore protein Nup98 in the pore complex and alter nucleocytoplasmic transport in the hippocampal neurons (Eftekharzadeh et al., 2018).

In terms of energy metabolism, the metabolite NAD^+^ and its associated histone deacetylase (HDAC) enzymes- sirtuins show significant decrease in aging and associated increased oxidative stress, causing catabolic breakdown of NAD^+^ (Braidy et al., 2011). Neuronal and axonal protection was achieved through increased NAD^+^ generation as well as downstream activation of the sirtuins, SIRT1 (Bedalov and Simon, 2004). These characteristics are affected in neurodegenerative diseases such as Alzheimer’s and Parkinson’s (Raff et al., 2002). NAD^+^ depletion in aging could be due to PARP [poly (ADP-ribose) polymerase], which has been reported to show increased expression in cells from Alzheimer’s and Parkinson’s patients (Cosi et al., 1996; Love et al., 1999; Bürkle et al., 2004). On the genetic level, brain aging attributes certain cognition specific changes. Cognition in terms of synaptic plasticity and memory are regulated by the expression of certain immediate-early genes such as *arc*, *bdnf*, *zif268*, *c-fos*, and *Egr1* (Penner et al., 2010; Gallo et al., 2018). Most of the immediate early genes are involved to be regulating functions of Ca^2+^ regulation, myelin turnover, vesicular transport, synaptic plasticity as well as energy metabolism and they are known to decrease in transcription with aging (Blalock et al., 2003). Reduced expression of these genes in adult brain hampers memory consolidation and occurs in normal aging as well as memory disorders (Linnarsson et al., 1997; Rosi et al., 2005; Rowe et al., 2007).

### Changes at the Epigenetic Level

A longitudinal study on a sample size of 104, performed with data on episodic memory as a parameter for tests, showed that the epigenetic DNA-methylation age predicted dementia significantly when compared to the chronological age (Degerman et al., 2017). The same study has also shown associations with the epigenetic age with cognitive impairment, deteriorating working memory. The group that displayed maintained cognition at an older age of 70–80 had a younger epigenetic age when compared to the baseline levels of those at the age of 55–65. Apart from modifications that happens during gene expression, learning and memory are said to be driven by an “epigenetic code.” Changes in this could lead to cognitive impairment particularly in learning and memory. Various signature patterns have been observed to be involved in the behavior patterns and these are the behavioral changes that are seen to be prominent in the process of aging (see Table 3). It is highly likely that the epigenetic landscape involved in such behavioral aspects of learning and memory is affected with aging.

**TABLE 3:** Prominent behavior changes in aging and underlying epigenetic code.

Epigenetic code/modification	Genes affected	Behavior changes/cognitive parameter affected	References
DNA cytosine methylation (MeC)	*PP1; reelin; BDNF; calcineurin; Arc; Egr1; Fos; Homer1; Nr4a2*	Conditioned fear memory; long-term associative memory formation and consolidation	Miller and Sweatt (2007), Bekinschtein et al. (2008), Lubin et al. (2008), Miller et al. (2010), Kaas et al. (2013)
Cytosine hydroxymethylation (OHMeC)	*Tdg; Apobec1; Smug; Mbd4*	Long-term associative memory formation and consolidation	Kaas et al. (2013)
H3 phosphorylation at Ser 10 and acetylation at K14	Not mentioned	Conditioned fear memory- long-term memory consolidation	Chwang et al. (2007)
H4 acetylation at K12	*Fmn2*	Associative learning, conditioned fear memory	Peleg et al. (2010)
H3 and H4 acetylation	*Nr4a1; Nr4a2; NGFI-B*	Contextual fear conditioned memory	Vecsey et al. (2007)
H3 acetylation at K9 and H4 acetylation	Not mentioned	Spatial learning and memory	Castellano et al. (2012)
H2B acetylation at Lys 5, 12, 15, 20 and H4 acetylation at Lys 12	*bdnf, cFos, FosB* and *zif268*	Spatial memory and consolidation	Bousiges et al. (2010)

DNA methylation is one among the epigenetic regulators of gene expression and is controlled through family of enzymes, DNMTs (DNA methyl transferases). They take part in establishing spatial memory and also in fear conditioning (Miller and Sweatt, 2007; Feng et al., 2010). DNMTs catalyze the methylation of the nucleotide cytosine at its 5th carbon to form 5-methyl cytosine (5mC). These 5mC are usually found in CpG dinucleotides in stretches of DNA termed “CpG islands.” The mammalian brain shows approximately 62% methylated CpGs (Fasolino and Zhou, 2017). These islands have been studied to regulate gene expression through mechanisms such as CpG methylation (Kotambail et al., 2017). DNMTs rely on methyl group donors such as L-methylfolate via SAM (Stahl, 2010), however, there is deficiency of folate observed in aging as well as cases of dementia (Robins Wahlin et al., 2001; Reynolds, 2002), parallel to the decrease in global methylation with aging. Increased methylation at the *PP1* gene and decreased methylation at the *reelin* gene underlie conditioned fear memory (Miller and Sweatt, 2007). Similarly, *BDNF* has been studied to be strongly linked to enduring fear memories through the suppression of its fourth exon by promoter methylation (Alonso et al., 2005; Bekinschtein et al., 2008). While methylation at promoters of first and sixth exons led to increased transcription of the gene (Lubin et al., 2008). These changes are reversed within 24 h and occur in the hippocampus where memory formation occurs. However, memory storage in the prefrontal cortex was coinciding with persistent methylation at the CpG rich promoter of *calcineurin* (Miller et al., 2010). These studies showed that treatment with DNMT inhibitors at the hippocampus gave mixed results due to the dual role of methyl adding and removing activities whereas treatment at the prefrontal cortex prevented retrieval of the fear memory. These modifications also interact and lead to other epigenetic regulators such as HDACs to bring about suppression of the genes. Apart from methylation, hydroxymethyl cytosine formed by the oxidative environment as well as through the catalytic activity of methylcytosine dioxygenase TET1 (Jessop and Toledo-Rodriguez, 2018), is known to bring about changes in the expression of certain genes such as *Tdg, Apobec1, Smug*, and *Mbd4*. This has been studied to affect long-term associative memory, its formation as well as consolidation (Kaas et al., 2013). Chromatin remodeling occurs through covalent modifications to the histone core proteins- acetylation, phosphorylation in establishing long-term memory. Mitogen- and stress-activated protein kinase-1 (MSK1) is a major phosphorylating kinase that target H3 histones and other signaling molecules such as cAMP response element-binding protein (CREB) and other transcription factors involved in synaptic plasticity and memory (Deak et al., 1998; Soloaga et al., 2003). MSK1 shows high expression in the hippocampus and the double knockout models lack in long-term contextual fear memory but are not affected in terms of short-term associative memory of fear. These models also show a deficit in spatial learning and display an impairment in passive avoidance learning. The kinase acts on ERK to regulate histone modifications post fear training- H3 phosphorylation at Ser 10 and acetylation at Lys 14, which indicate transcriptional activation (Chwang et al., 2007).

Upregulated histone acetylation post-treatment with HDAC inhibitors has been shown to enhance memory formation and LTP (Levenson et al., 2004). Other similar studies pertaining to histone acetylation have been shown to be linked to activated gene expression of those that regulate cognition pertaining functions such as increased hippocampal synaptic connectivity/plasticity, LTP (Levenson et al., 2004; Vecsey et al., 2007; Peleg et al., 2010). Acetylation at H3 and H4 are generally linked with associative learning, conditioned fear memory- long-term storage and consolidation which have been studied to decrease with aging (Chwang et al., 2007; Vecsey et al., 2007; Peleg et al., 2010). These modifications usually occur at promoter regions of genes such as *Fmn2* (formin 2), *Nr4a1*, *Nr4a2* (nuclear receptor subfamily 4 group A member 1 and 2), *NGFI-B* (nerve growth factor I-B) which are known to take part in regulation of cytoskeletal structures, LTP, hippocampal dependent memory storage. Similar study revealed a multiple background changes in the epigenome with aging and highlighted that with aging there is also a region-wise change. It showed H3 acetylation at Lys 9 to be decreased in hippocampal CA1 region, no changes in DG (dentate gyrus) or CA3, whereas, H4 acetylation showed an opposite regulation with aging, increased in CA1 and reduced in DG. These changes were noted as with normal aging accompanied cognitive impairment in terms of spatial learning and memory (Castellano et al., 2012). H2B acetylation at Lys 5, 12, 15, 20, and H4 acetylation at Lys 12 affect spatial memory and its consolidation. These changes affect genes such as *bdnf, cFos, FosB*, and *zif268* (Bousiges et al., 2010).

Oxidative stress in form of ROS induced DNA lesions can influence and alter the methylome landscape in a cell, by means of DNA oxidation and TET-mediated hydroxymethylation (Kriaucionis and Heintz, 2009; Tahiliani et al., 2009). These lesions are pyrimidine hydroxylation products and 5-methylcytosine (5mC). They could interfere with the actual 5-hmC signals by their structural similarities (Lewandowska and Bartoszek, 2011). DNA lesions such as 8-oxoguanine could be repaired through mechanisms of base excision repair (BER) through enzyme 8-oxoG glycosylase (OGG1), however, it has also been shown to be involved in demethylation of methyl-CpGs from 5mC to cytosine, this could lead to changes in the epigenetic signatures of the genes and hence their expression (Zhou et al., 2016). CpG sites demethylation plays roles in memory formation as well as consolidation in hippocampus and the cingulate cortex (Duke et al., 2017). Oxidative stress could also lead to oxidation followed by deamination of the oxidized 5mC (Zuo et al., 1995). Cytosine and 5-methylcytosine give rise to varied products under the stress of ROS such as hydroxyl anions (see Table 1). Oxidation of cytosine would be a major factor as it enables its deamination to bring about GC → AT transitions.

Studies showed that hippocampal cells from Alzheimer’s patients had decreased global methylation as well as hydroxymethylation (Chouliaras et al., 2013). Oxidative stress has the ability to alter methylation and histone acetylation, and hence may be a determinant regulatory factor that affects the epigenetics of cells (Gu et al., 2013). Another example of a reactive oxidant species regulating epigenetics of the aging brain is nitric oxide (NO). NO at appropriate concentrations reported to be neuroprotective; however, at excess concentrations, it has been shown to react with superoxide to form the highly reactive peroxynitrite contributing to the oxidative stress (Radi, 2013; Dubey et al., 2017). Sirt1 is known to regulate the production of NO *via* the acetylation of endothelial nitric oxide synthase (eNOS) (Donato et al., 2011; Martins, 2017b). The age associated decrease in Sirt1 seems to bring about deregulation of NO synthesis which in turn has a profound impact on various downstream targets of NO including DNA methylation, histone acetylases as well as histone methyltransferases (Socco et al., 2017). NO has been shown to have intricate connections with *BDNF*, which is involved in cognition (learning, memory, and its consolidation), synaptic plasticity, and LTP (Paul and Ekambaram, 2011; Kolarow et al., 2014; Dubey et al., 2017). *BDNF* regulates post-synaptically the production of NO in the hippocampal neurons at the dendrites and the soma through TrkB and TrkC based signaling (Kolarow et al., 2014). Reduced *BDNF* levels in the brain has been studied in the context of downstream result of deregulated NO synthesis, and also resulting cognitive impairment (Canossa et al., 2002; Banoujaafar et al., 2016). In the cortex and the hippocampus, NO shows gradual decline with aging (Reckelhoff et al., 1994; Lima-Cabello et al., 2016). Clinical samples of blood plasma in aged individuals have been studied to show higher levels of NO and has been implicated to loss of auditory-verbal as well as visual-spatial based working memory along with impairment of short term declarative memory (Talarowska et al., 2012). Aβ protein disrupts NO activity while impairing the synapses and LTP in Alzheimer’s disease (Paul and Ekambaram, 2011).

**FIGURE 1:**
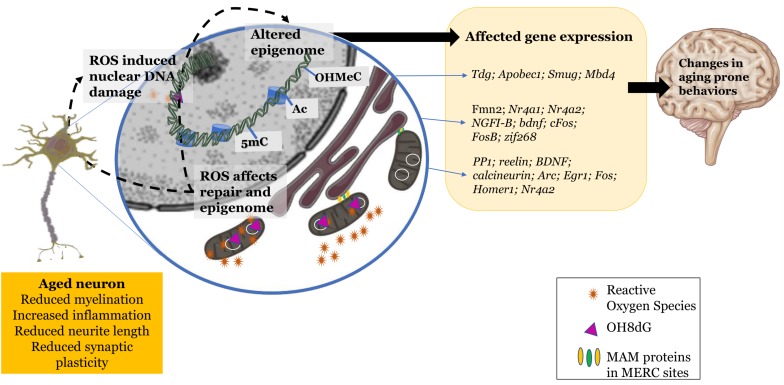
Changes exhibited by the aging neuron: increased reactive oxidant species production, mitochondrial and nuclear DNA damage, dysfunctional mitochondrial-endoplasmic reticular sites. These changes in the redox environment in the cytosol as well as nucleus trigger epigenetic changes leading to altered gene expression and further leading to changes in aging prone behaviors. Schematic parts of the figure were taken from Servier Medical art (https://smart.servier.com/) licensed under an Unported license of Creative Commons Attribution 3.0.

The oxidative environment does seem to play an influence in regulating epigenetic machinery and the resulting epigenome of the aging cell. This affects certain characteristics of the cellular system in terms of plasticity and transmission efficacy that ultimately alter cognition to become prone to a gradual decline (Figure 1).

## Evolving Theories of Aging

The long-established theory that was made to attempt explaining the mechanism underlying aging explains it as the accumulating cellular and molecular damage through reactive oxidant species, is well known as the Free radical theory (Harman, 1956). There are now several theories as well as updated versions to this theory with better explanations to the demerits. One among them which is finding favors among many in the field of aging research is the damage theory. A strong argument that the damage theory poses is that the although antioxidants such as mitochondrial SOD seem to ameliorate conditions that prevail in oxidative stress situations, they don’t seem to extend the life span through overexpression (Mockett et al., 2010). A recent theory that explains the mechanisms that prevail during aging is the epigenetic oxidative redox shift (EORS) theory. It attempts to explain the shift in metabolism with aging. It states the probable increased glycolysis that is brought about through the impaired mitochondrial system, which would ultimately result in increased ROS production. This is carried out by the upregulation of the redox-sensitive transcription factors. The upstream shift is said to be toward oxidized metabolic shift (Jones, 2006) which is due to reduced bursts of energy requirement that is resultant of sedentary lifestyle or lower physical activity. The shift is epigenetically regulated through chromatin modulators - HDACs such as sirtuins, histone acetylases as well as DNMTs. This is accompanied by insulin resistance (Brewer, 2010).

## Molecular Targets - A Strategy Aiming for Therapy

The process of oxidative damage has opened avenues for probable targets that either aid in apoptotic inhibition, reducing ROS, modulate chromatin architecture to keep learning and memory associated genes active in transcription. Some of them have been listed below. Quinone reductase 2 is one among the many genes that undergo changes in their hippocampal expression pattern with aging. Its overexpression is reported to be involved in learning deficits that occur in the case of age-related memory impairment. Its selective inhibitors, S26695 and S29434, were able to protect against the toxin menadione-induced apoptosis, preserving and enhancing learning abilities. Similarly, knockout models showed improved motor learning skills (Benoit et al., 2010).

Human TFAM protein was overexpressed in transgenic mice which suppressed ROS sourced from the mitochondria as well as the inflammatory IL-1β response. It increased the mean EPSP (excitatory postsynaptic potential) when compared to the wild-type aged mice, it ameliorated the working memory as well the hippocampal LTP (Hayashi et al., 2008). Treatment with spin-trapping compound N-*tert*-butyl-α-phenylnitrone (PBN) helped to cut down the increasing reserves of ROS and prevented early loss of glutamine synthetase activity. The treatment also reduced error rate in radial arm tests indicating that cognitive dysfunction does result from the accumulation of oxidant species, oxidized proteins and products; this impairment in cognition induced through oxidative stress is rescued through the treatment with spin trap moieties, however, this was later observed to be species specific (Carney et al., 1991). Epigenetic mechanisms such as histone deacetylation could be inhibited *via* HDAC inhibitors such as suberoylanilide hydroxamic acid (SAHA). This inhibits class I HDACs and has been observed to induce expression of learning and memory genes in aged mice (Peleg et al., 2010). The same compound along with other HDAC inhibitors such as sodium valproate and sodium butyrate have been studied to rescue the long-term memory deficits in case of contextual memory in Alzheimer’s disease mice models (Kilgore et al., 2010). HDACI I2 and W2 brought about increased mRNA levels of Aβ degradation enzymes as well as decreased Aβ levels and rescued learning and memory deficits in aged mice Alzheimer’s model- hAPP 3x Tg AD. The HDACI W2 also decreased tau phosphorylation at amino acid position threonine-181 (Sung et al., 2013).

Another possible target which could also bring about epigenetic regulation through HDAC sirtuins is NAD^+^. NAD^+^ is essential as a cofactor, a hydride donor in several metabolic functions necessary for cell’s survival, a key player in energy metabolism- glycolysis, tricarboxylic acid cycle, mitochondrial oxidative phosphorylation (OXPHOS) as well as fatty acid β-oxidation (Wallace, 2012; Fang et al., 2017). NAD^+^ serves as a substrate for SIRT1, to perform its gene regulatory function. Increased levels of NAD^+^ has been shown to promote more effective SIRT1 activity (Araki et al., 2004; Milne and Denu, 2008; Massudi et al., 2012b). NAD^+^ is seen to be affected with chronic exposure to oxidative stress, which leads to its catabolism (Furukawa et al., 2007; Massudi et al., 2012a). SIRT1 correspondingly showed decreased activity with progressing age (Braidy et al., 2011).

On the other hand, Sirt1 levels could be regulated by means of certain activators and inhibitors (such as bacterial lipopolysaccharides-LPS). Its activators such as resveratrol have applications in stabilizing cases of epilepsy as well as epilepticus (Martins, 2017a). This could be potential alternative in molecular therapeutic strategies.

L-Arginine as a donor of NO is considered for potential therapeutic application. It showed neuroprotective roles along with NO to bring about amelioration of age induced memory impairment (Paul et al., 2005; Paul and Ekambaram, 2011; Hosseini et al., 2012). It also attenuated oxidative stress as well as DNA damage in models of sporadic Alzheimer’s disease (Dubey et al., 2017).

### Future Direction: Toward Healthier Aging

**FIGURE 2:**
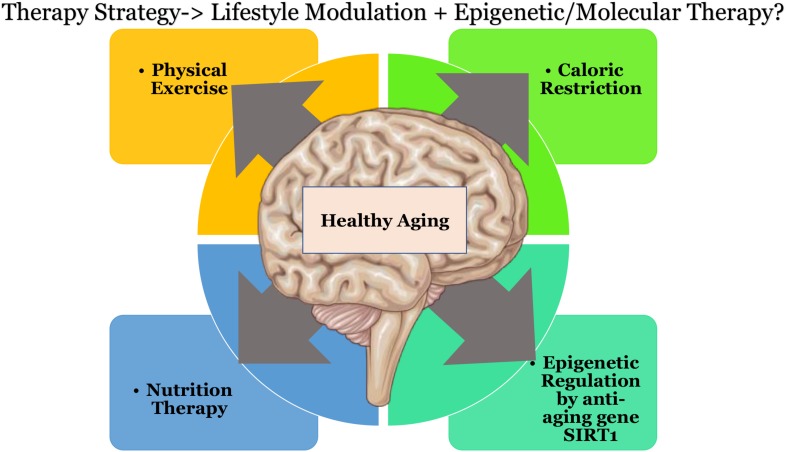
Possible therapeutic directions that could result in healthier aging of the brain. Schematic parts of the figure were taken from Servier Medical art (https://smart.servier.com/) licensed under an Unported license of Creative Commons Attribution 3.0.

Therapeutic strategies may be devised using multiple approaches to curb the effects of oxidative stress, this could be in the form of caloric restriction (Martins, 2017c) coupled with exercise. Physical exercise has been shown to increase the levels of blood flow in the brain vasculature and also to influence *BDNF* based neuroplasticity (Banoujaafar et al., 2016). Other approaches could involve nutrition therapy to supplement key players that help in epigenetic regulation with amino acids such as L-arginine, diet with Sirt1 activators, or downstream metabolites such as NAD^+^, or through quinone reductase inhibitors. Individual therapeutic strategies do not seem to show promising or effective results as there seems to exist a background interconnectivity in the treatment modules toward healthy aging of the brain, that would affect multiple pathways downstream (as shown in Figure 2). Keeping in mind that “we are what we eat” (Twiss, 2007) and that there is “no clear-cut definition of normal aging” (Vauzour et al., 2017) due to individual based differences. There could be substantial opportunities toward discovering pathways to healthy aging.

## Conclusion

When observing the cellular background of cognitive decline, various aspects come into the picture that seem to be the basis of pathogenesis- oxidative stress and its associated damage, vast changes in the metabolic landscape, epigenetic variations, all of which accompany organellar dysfunction and the shortfall in repair and recovery mechanisms. Most of the studies highlight the significant association between aging and oxidative stress; however, still more studies have to be conducted that can show a direct causal link between the two. There is an increasing number of studies that point toward the epigenetic regulation of cognition through several mechanisms that affect the genes involved in learning and memory. Such that there might be a histone code that is involved in the regulation of cognition that may be impacted during aging. All the studies converge to a point that highlight the presence of oxidative stress in the aging cells, cognition in particular is affected via various mechanisms at the gene, nucleocytoplasmic and mostly the epigenetic level. Each component could pose as targets for therapy for symptomatic therapy but for a holistic approach several strategies that also involve epigenetic machinery would prove to be ideal. There is a need for longitudinal studies *in vivo* and in humans to understand an overall perspective to understanding the placement of aging and its associated diseases. Longitudinal studies in aging animal models (natural and/or induced) and further into humans involving a combined therapeutic strategy of regulated lifestyle modules that incorporate the above-mentioned therapeutic concepts could help understand the fundamental pathology of aging and hence determine effective regulation of healthy aging. There also seems to be several lacunae in understanding the models (D-galactose induced, accelerated senescence, delayed aging, transgenic) to understand aging does not account for the complete pathology of aging (Mitchell et al., 2015). As aging has multiple confounding factors that play a role in determining the level of damage (multiple morbidities) (Santulli et al., 2015), it would be necessary to understand the combined effects of multiple factors to establish causal links between aging and oxidative stress and also the associated cognitive impairment.

## Author Contributions

AK synthesized and generally organized the manuscript, Figures 1, 2, and Tables 1–3. GG and KS supervised and edited the manuscript. AK declared that substantially contributed to the review article in object.

## Conflict of Interest

The authors declare that the research was conducted in the absence of any commercial or financial relationships that could be construed as a potential conflict of interest.
